# Perspectives on the Use of Multiple Sclerosis Risk Genes for Prediction

**DOI:** 10.1371/journal.pone.0026493

**Published:** 2011-12-02

**Authors:** Naghmeh Jafari, Linda Broer, Cornelia M. van Duijn, A. Cecile J. W. Janssens, Rogier Q. Hintzen

**Affiliations:** 1 Department of Neurology, ErasMS Center, Erasmus MC, Rotterdam, The Netherlands; 2 Department of Epidemiology, Erasmus MC, Rotterdam, The Netherlands; Innsbruck Medical University, Austria

## Abstract

**Objective:**

A recent collaborative genome-wide association study replicated a large number of susceptibility loci and identified novel loci. This increase in known multiple sclerosis (MS) risk genes raises questions about clinical applicability of genotyping. In an empirical set we assessed the predictive power of typing multiple genes. Next, in a modelling study we explored current and potential predictive performance of genetic MS risk models.

**Materials and Methods:**

Genotype data on 6 MS risk genes in 591 MS patients and 600 controls were used to investigate the predictive value of combining risk alleles. Next, the replicated and novel MS risk loci from the recent and largest international genome-wide association study were used to construct genetic risk models simulating a population of 100,000 individuals. Finally, we assessed the required numbers, frequencies, and ORs of risk SNPs for higher discriminative accuracy in the future.

**Results:**

Individuals with 10 to 12 risk alleles had a significantly increased risk compared to individuals with the average population risk for developing MS (OR 2.76 (95% CI 2.02–3.77)). In the simulation study we showed that the area under the receiver operating characteristic curve (AUC) for a risk score based on the 6 SNPs was 0.64. The AUC increases to 0.66 using the well replicated 24 SNPs and to 0.69 when including all replicated and novel SNPs (n = 53) in the risk model. An additional 20 SNPs with allele frequency 0.30 and ORs 1.1 would be needed to increase the AUC to a slightly higher level of 0.70, and at least 50 novel variants with allele frequency 0.30 and ORs 1.4 would be needed to obtain an AUC of 0.85.

**Conclusion:**

Although new MS risk SNPs emerge rapidly, the discriminatory ability in a clinical setting will be limited.

## Introduction

Multiple sclerosis (MS) is caused by an interplay of multiple genetic variants and environmental factors. The genetic influence on MS is substantial, as evidenced by the 20-fold risk increase for siblings of MS patients [1]. Part of the genetic risk is explained by the MHC class II locus (*HLA-DR15*) [2]. In 2007 several novel risk alleles for MS were identified by a genome-wide association (GWA) study [3] and others confirmed the susceptibility loci by meta-analyses and replication [4]. Since GWA the progress has been rapid and more new risk loci have been identified and confirmed [5], [6], [7], [8], [9]. A recent study in 9,722 cases and 17,376 controls identified 53 associated variants [9].

Given the gene-environmental and multi-genetic causes of MS, these susceptibility variants mainly have weak effects and are likely to contribute to a small increase in MS risk individually. It is commonly agreed that testing single susceptibility genes is not useful for prediction of MS risk, but the question remains whether combining susceptibility loci in risk models could have an added value on MS prediction in individuals. The predictive performance of genetic risk models has been investigated for other diseases in simulation studies [10], [11]. These studies suggest that the predictive value improves by combining multiple common low-risk loci.

We investigated the extent to which MS risk can be predicted using genetic risk models. First of all we tested in our empirical data the predictive performance of 6 combined genotyped SNPs, using risk scores compared to a prior chance of someone in our population having MS. However whether genetic risk models will potentially be used in clinical or public health practices depends on the accuracy of the test to discriminate between individuals who will develop MS and who will not. The discriminative accuracy is generally expressed as the area under the receiver operating characteristic curve (AUC). Therefore, secondly we tested the potential performance of SNP genotyping in a simulation study by adding risk genes into the model. For this, we constructed a risk model based on 1) the 6 genotyped SNPs, 2) the 24 recently well replicated genome-wide associated polymorphisms [9] and 3) the 53 replicated genome-wide associated polymorphisms including the 29 newly identified polymorphisms [9]. Finally, we included hypothetical variants in the risk model, in order to investigate the future potential.

## Methods

### Empirical study

#### Ethics Statement

This study was approved by the Ethics Committee of the Erasmus University Medical Centre, METC Erasmus MC Rotterdam. All participants were recruited in Erasmus University Medical Centre and written informed consent was obtained.

#### Study population

A total of 591 MS patients and 600 controls were included in this study. The MS patients were recruited and ascertained as part of an ongoing nationwide study on genetic susceptibility in MS and fulfilled McDonald criteria for MS [12]. Details on ascertainment are given elsewhere [13].

#### Genotyping

The *HLA-DRB* rs3135388, *EVI5* rs10735781, *CLEC16A* rs64981169, *CD58* rs12044852, *IL7R* rs6897932, and *IL2RA* rs2104286 SNPs (table 1) were genotyped using the MassARRAY system/Homogeneous MassExtend assay, following the protocol provided by Sequenom. PCR extension primers were designed using the Assay Design 3.0 program (Sequenom). ThermoSequenase (Sequenom) was used for the base extension reactions. Analysis and scoring were performed using the program Typer 3.3 (Sequenom).

**Table 1:** Individual association of 6 genotyped SNPs in the empirical study.

			*Controls*		*Cases*			
Gene	Variant	Risk Allele	n genotyped	RAF	n genotyped	RAF	OR (95% CI)	p-value
*HLA-DRB*	rs3135388	T	599	0.14	588	0.28	2.53 (2.02–3.17)	8.14*10^−16^
*EVI5*	rs10735781	G	597	0.33	586	0.38	1.19 (1.01–1.42)	0.044
*CLEC16A*	rs64981169	G	593	0.33	583	0.39	1.27 (1.07–1.51)	0.006
*CD58*	rs12044852	C	599	0.88	587	0.91	1.50 (1.14–1.97)	0.004
*IL7R*	rs6897932	C	599	0.72	588	0.76	1.21 (1.00–1.46)	0.045
*IL2RA*	rs2104286	A	595	0.73	581	0.76	1.14 (0.95–1.38)	0.157

RAF: risk allele frequency, OR: odds ratio, 95% CI: 95% confidence interval.

#### Risk score analysis

All statistical analyses on empirical data were performed using SPSS version 15. Associations of individual SNPs were investigated using logistic regression. We also applied logistic regression analyses to investigate the combined predictive value of the risk allele score based on all SNPs with and without *HLA-DRB* (rs3135388) using the *a priori* probability of an individual in our population developing MS as reference. As we tested a total of 6 SNPs in our empirical study, the Bonferoni-corrected p-value for significance was 0.008. The weighted risk allele score was calculated by multiplying the number of risk alleles with the effect size for each SNP obtained from the literature and summing this up for each participant with complete genotype information, with risk alleles being the alleles associated with increased risk of MS. All analyses were adjusted for age and sex.

### Simulation study

#### Modelling strategy

We used a modelling procedure that has been developed and published previously [14], and which has also been used by others [15]. Briefly, the procedure creates a dataset with information on genotypes and disease status for a population of 100,000 individuals. The dataset is constructed in such a way that the odds ratios and frequencies of the genotypes and the disease risk match the specified values, which are obtained from the literature. Predicted MS risks are calculated using Bayes' theorem, which states that the posterior odds of MS for each individual is obtained by multiplying the prior odds by the likelihood ratio (LR) of their genotype status on all polymorphisms. The prior odds is calculated from the baseline population MS risk (p) using the formula p/(1-p). Under the assumption of independent genetic effects i.e., no linkage disequilibrium between the genetic variants, the LR is obtained by multiplying the LRs of all individual genotypes that are included in the risk model [16]. The LRs of the genotypes of each single genetic variant are calculated from a genotype by disease status contingency table [14]. This table is constructed from the frequency and ORs of the genotypes and the population MS risk. The table can also be constructed from allele frequencies and per allele ORs when Hardy-Weinberg Equilibrium is assumed for the distribution of the genotypes. The frequencies and ORs all are specified as study parameters and varied between the simulation scenarios. The posterior odds are converted into MS risks using the formula odds/(1+odds).

#### Discriminative accuracy

The discriminative accuracy is the extent to which the test results can discriminate between individuals who will develop MS and those who will not [17]. The AUC gives an assessment of the discriminative accuracy of a prediction model and ranges from 0.5 (equal to tossing a coin) to 1.0 (perfect prediction). All simulations were repeated 100 times to obtain robust estimates of the AUC. All results are presented as averages of the repeated simulations. The obtained confidence intervals were extremely small, often equal to the point estimate, and therefore not presented in this paper. Analyses were performed using R software (version 2.12.1) [18].

#### Simulation scenarios

Recently, a large GWA study was presented as part of the collaboration between Wellcome Trust Case Control Consortium 2 (WTCCC2) and the International Multiple Sclerosis Genetics Consortium (IMSGC) [9]. Twenty-three MS associated non- major histocompability complex (MHC) loci were replicated in the primary GWAS involving 9,772 cases and 7,296 controls with P_GWAS_<1*10^−3^. Table 2 provides the 23 replicated non-MHC SNPs with the combined ORs and p-value. The risk allele frequency represents the allele frequency in control population of UK, as being the largest sample. Table 2 also includes the HLA-DRB1*15∶01 MHC SNP, which have been shown to significantly increase the risk for MS. These 24 risk SNPs also include the 6 polymorphisms of our empirical data. The collaboration also presented the identification of 29 novel susceptibility loci as shown in table 3. This leads to a total of 53 risk SNPs.

**Table 2:** Summary of the 24 replicated multiple sclerosis associated risk loci.

*Gene*	*Variant*	*Chromosome*	*Risk allele*	*RAF*	*OR* (95% CI)	*P-value*
*MMEL1*	rs4648356	1	C	0.67	1.14 (1.12–1.16)	1.00*10^−14^
*EVI5*	rs11810217	1	A	0.25	1.15 (1.13–1.16)	5.80*10^−15^
*CD58*	rs1335532	1	A	0.87	1.22 (1.19–1.24)	3.20*10^−16^
*RGS1*	rs1323292	1	A	0.83	1.12 (1.10–1.14)	2.30*10^−8^
*KIF21B*	rs7522462	1	G	0.70	1.11 (1.10–1.13)	1.90*10^−9^
*CBLB*	rs2028597	3	G	0.91	1.13 (1.06–1.21)	2.10*10^−4^
*TMEM39A*	rs2293370	3	G	0.80	1.13 (1.11–1.15)	2.70*10^−9^
*IL12A*	rs2243123	3	G	0.29	1.08 (1.06–1.10)	7.20*10^−6^
*IL7R*	rs6897932	5	G	0.73	1.11 (1.09–1.13)	1.70*10^−8^
*PTGER4*	rs4613763	5	G	0.13	1.20 (1.18–1.22)	2.50*10^−16^
*HLA-DRB*	rs3135388	6	A	0.13	3.08 (not shown)	<1.0*10^−320^
*OLIG3*	rs13192841	6	A	0.27	1.10 (1.09–1.12)	1.30*10^−8^
*IL7*	rs1520333	8	G	0.25	1.10 (1.08–1.11)	1.60*10^−7^
*IL2RA*	rs3118470	10	G	0.32	1.12 (1.10–1.13)	3.20*10^−11^
*ZMIZ1*	rs1250550	10	A	0.35	1.10 (1.09–1.12)	6.30*10^−9^
*CD6*	rs650258	11	G	0.63	1.12 (1.10–1.13)	2.00*10^−11^
*TNFRSF1A*	rs1800693	12	G	0.40	1.12 (1.11–1.14)	4.10*10^−14^
*CYP27B1*	rs12368653	12	A	0.47	1.10 (1.09–1.12)	1.70*10^−9^
*MPHOSPH9*	rs949143	12	G	0.28	1.08 (1.04–1.12)	1.50*10^−4^
*CLEC16A*	rs7200786	16	A	0.46	1.15 (1.13–1.16)	8.50*10^−17^
*IRF8*	rs13333054	16	A	0.23	1.11 (1.10–1.13)	1.30*10^−8^
*STAT3*	rs9891119	17	C	0.36	1.11 (1.09–1.12)	1.80*10^−10^
*TYK2*	rs8112449	19	G	0.67	1.08 (1.07–1.10)	1.20*10^−6^
*CD40*	rs2425752	20	A	0.25	1.11 (1.10–1.13)	5.10*10^−10^

RAF: risk allele frequency, OR: odds ratio, 95% CI: 95% confidence interval.

OR and p-value represent the combined discovery and replication study results [9]. Risk allele frequency refers to allele frequency in control population of UK samples. For CBLB is the discovery OR and p-value given.

Reprinted by permission from Macmillan Publishers Ltd: The International Multiple Sclerosis Genetics Consortium and The Wellcome Trust Case Control Consortium. Genetic risk and a primary role for cell-mediated immune mechanisms in multiple sclerosis. 2011, Nature 476: 214–219.

**Table 3:** The 29 novel associated MS risk genes.

*Gene*	*Variant*	*Chromosome*	*Risk allele*	*RAF*	OR OR (95% CI)	*P-value*
*VCAM1*	rs11581062	1	G	0.29	1.12 (1.10–1.13)	2.50*10^−10^
*No gene*	rs12466022	2	C	0.73	1.11 (1.10–1.13)	6.20*10^−10^
*PLEK*	rs7595037	2	A	0.55	1.11 (1.10–1.12)	5.10*10^−11^
*MERTK*	rs17174870	2	G	0.75	1.11 (1.09–1.13)	1.30*10^−8^
*SP140*	rs10201872	2	A	0.18	1.14 (1.12–1.16)	1.80*10^−10^
*No gene*	rs669607	3	C	0.48	1.13 (1.12–1.15)	1.90*10^−15^
*EOMES*	rs11129295	3	A	0.36	1.11 (1.09–1.12)	1.20*10^−9^
*CD86*	rs9282641	3	G	0.91	1.21 (1.18–1.24)	1.00*10^−11^
*IL12B*	rs2546890	5	A	0.52	1.11 (1.10–1.13)	1.20*10^−11^
*BACH2*	rs12212193	6	G	0.47	1.09 (1.08–1.10)	3.80*10^−8^
*THEMIS*	rs802734	6	A	0.69	1.10 (1.09–1.12)	5.50*10^−9^
*MYB*	rs11154801	6	A	0.36	1.13 (1.11–1.15)	1.00*10^−13^
*IL22RA2*	rs17066096	6	G	0.24	1.14 (1.12–1.15)	6.00*10^−13^
*TAGAP*	rs1738074	6	G	0.57	1.13 (1.12–1.15)	6.80*10^−15^
*ZNF746*	rs354033	7	G	0.74	1.11 (1.10–1.13)	4.70*10^−9^
*MYC*	rs4410871	8	G	0.72	1.11 (1.09–1.12)	7.70*10^−9^
*PVT1*	rs2019960	8	G	0.23	1.12 (1.10–1.13)	5.20*10^−9^
*HHEX*	rs7923837	10	G	0.62	1.10 (1.08–1.11)	4.90*10^−9^
*CLECL1*	rs10466829	12	A	0.50	1.09 (1.08–1.11)	1.40*10^−8^
*ZFP36L1*	rs4902647	14	G	0.53	1.11 (1.10–1.13)	9.30*10^−12^
*BATF*	rs2300603	14	A	0.74	1.11 (1.09–1.12)	2.00*10^−8^
*GALC*	rs2119704	14	C	0.92	1.22 (1.19–1.25)	2.20*10^−10^
*MALT1*	rs7238078	18	A	0.77	1.12 (1.10–1.14)	2.50*10^−9^
*TNFSF14*	rs1077667	19	G	0.79	1.16 (1.14–1.18)	9.40*10^−14^
*MPV17L2*	rs874628	19	A	0.72	1.11 (1.09–1.12)	1.30*10^−8^
*DKKL1*	rs2303759	19	C	0.25	1.11 (1.09–1.13)	5.20*10^−9^
*CYP24A1*	rs2248359	20	G	0.61	1.12 (1.10–1.13)	2.50*10^−11^
*MAPK1*	rs2283792	22	C	0.52	1.10 (1.08–1.11)	4.70*10^−9^
*ODF3B*	rs140522	22	A	0.33	1.10 (1.09–1.12)	1.70*10^−8^

RAF: risk allele frequency, OR: odds ratio, 95% CI: 95% confidence interval.

OR and p-value represent the combined discovery and replication study results [9]. Risk allele frequency refers to allele frequency in control population of UK samples.

Reprinted by permission from Macmillan Publishers Ltd: The International Multiple Sclerosis Genetics Consortium and The Wellcome Trust Case Control Consortium. Genetic risk and a primary role for cell-mediated immune mechanisms in multiple sclerosis. 2011, Nature 476: 214–219.

Three different simulation scenarios were considered. In each scenario genotypes and MS status were simulated for 100,000 individuals, assuming a lifetime MS risk of 0.1%. The first scenario calculated the AUC within the empirical data weighted on literature frequency. The second scenario assessed the increase in AUC by adding additional risk alleles, starting with the 6 genotyped risk loci given the replicated ORs. We compared this to the calculated AUC for validation of the simulation model. Next, the AUC was calculated with the 24 replicated SNPs in the recent Nature paper including the 6 genotyped SNPs. And finally, the AUC was assessed on a risk model including the 29 novel susceptibility loci on top of the replicated SNPs, leading to a total of 53 SNPs. The third scenario investigated the magnitude of the allele ORs of 1 to 100 polymorphisms that need to be added to the risk model to increase the discriminative accuracy. Since there are no models known in the literature for predicting MS risk we pursued AUCs known to be used for other diseases in the literature [19], [20]. We investigated AUC thresholds of 0.70, 0.75, 0.80 and 0.85. The ORs were obtained for different frequencies of the risk alleles.

## Results

### Empirical study

A total of 588 cases and 599 controls were successfully genotyped for at least one polymorphism, while complete genotype information on all polymorphisms was available for 564 cases and 581 controls. The mean age (SD) within the cases and controls was 45 (12) and 49 (17) years, respectively. The cases included 71% female and the controls 55%. None of the polymorphisms deviated significantly from Hardy Weinberg Equilibrium (lowest Hardy Weinberg p-value = 0.15 for *IL2RA*: rs2104286).

Table 1 shows the individual effects of each SNP on MS risk in our genotyped population. Increased risk for MS was confirmed for the minor alleles of *EVI5*, *HLA-DRB* and *CLEC16A*, and for the major alleles of *CD58* and *IL7R.* For *IL2RA* the association was not statistically significant (OR 1.14, 95% CI 0.95–1.38). When adjusting for multiple testing only *HLA-DRB*, *CLEC16A* and *CD58* remained statistically significant.

**Figure 1:**
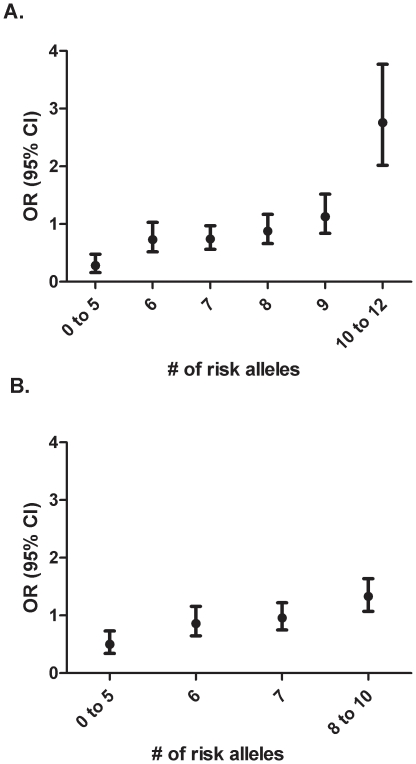
Weighted Risk scores for the 6 genotyped SNPs. The odds ratios for MS are shown according to the number of risk alleles carried. The reference value is based on the a priori probability of someone in the general population to carry MS risk alleles. A) Weighted risk scores for the 6 genotyped SNPs including *HLA-DRB*. B) Weighted risk scores for the 5 genotyped SNPs.

Figure 1A shows the risk score when including all SNPs into the model. The reference category is based on the *a priori* risk for developing MS, which in our population was 49% ( = 564 cases divided by 581 controls). Individuals with 0 to 5 risk alleles have a significantly decreased risk for developing MS of 0.28 (95% CI 0.16–0.48) compared to the *a priori* risk for developing MS. On the other end of the spectrum, individuals with 10 to 12 risk alleles have a significantly increased risk of 2.76 (95% CI 2.02–3.77). Figure 1B shows that, when excluding the variant with the strongest risk effect (*HLA-DRB*) from the risk score, individuals with 0 to 5 risk alleles have a decreased risk of 0.50 (95% CI 0.34–0.73) and individuals with 8 to 10 risk alleles have an increased risk of 1.33 (95% CI 1.07–1.64) in comparison with the *a priori* risk for developing MS.

### Simulation study

Table 2 provides the 24 replicated SNPs from the recent Nature paper [9] which have been shown to significantly increase the risk for MS. These 24 risk SNPs include also the 6 polymorphisms of our empirical data. Table 3 shows the 29 newly identified polymorphisms in this Nature paper, leading to a total of 53 risk SNPs.

**Figure 2:**
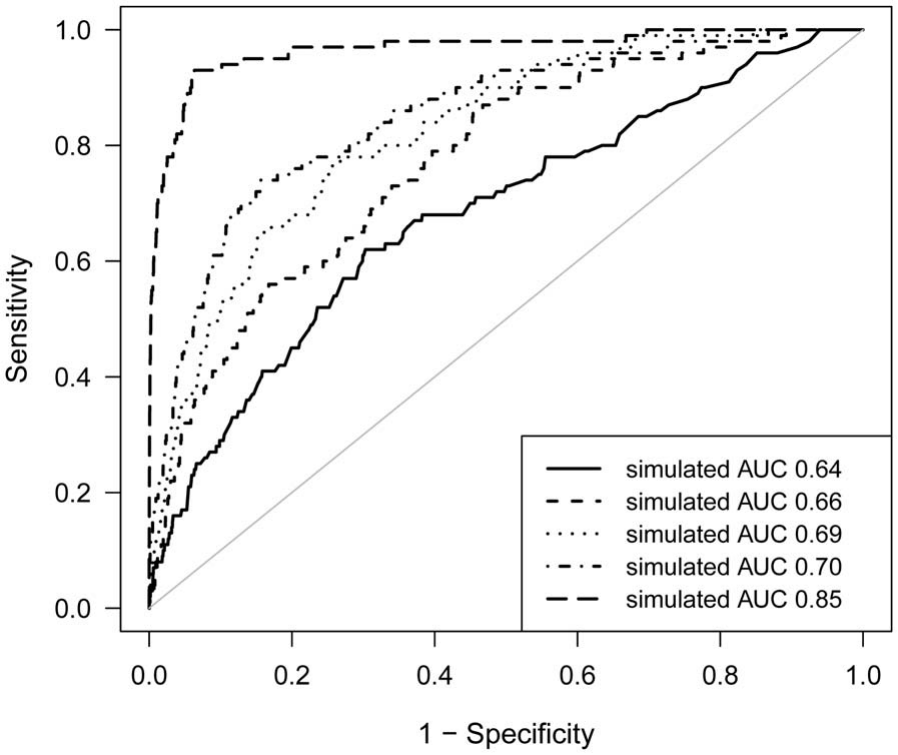
ROC curves for simulation models predicting MS. Four situations are depicted. Solid line (^____^) represents ROC curve for simulation model based on 6 genotyped SNPs (AUC 0.64). Dashed line (----) ROC curve for simulation model based on 24 well replicated SNPs (AUC 0.66). Dotted line (^……..^) ROC curve for simulation model based on a total of 53 replicated and novel SNPs (AUC 0.69). Dash-dotted line (^_^^.^^_^^.^) ROC curve for simulation model based on 20 extra variants with an arbitrarily set allele frequency of 0.30 and OR 1.1 (AUC 0.70). Long- dashed line (^__^ - ^__^) ROC curve for simulation model based on 50 extra variants with an arbitrarily set allele frequency of 0.30 and OR 1.4 (AUC 0.85).

First, we calculated that the AUC for the genotyped 6 SNPs within the empirical data weighted on literature frequency was 0.64. Second, in the simulation study we assessed the AUC increase by including additional risk alleles. The AUC for the recently replicated ORs of the 6 SNP's used in the empirical study was 0.64. This showed to be the same as the calculated AUC from the empirical study. Next, including the 24 known polymorphisms in the model the AUC rised to 0.66, and slightly increased to 0.69 after including all 53 SNPs in the model (Figure 2). Finally, we explored the possibilities in the future with new risk alleles to be discovered. Table 4 shows the number of new risk genes with specific allele frequencies in combination with different ORs that would be needed in addition to the original 53 risk variants to obtain AUCs of 0.70, 0.75, 0.80 and 0.85. For example to increase the AUC just slightly to 0.70 we have to add to our model 20 new variants, with a realistic OR of 1.1 and an allele frequency of 0.30. However if we want to increase the AUC to 0.85 we have to add 50 new variants with an OR of 1.4 and an allele frequency of 0.30. For more realistic ORs this would mean we would have to add even more polymorphisms to the model.

**Table 4:** Odds ratios and related allele frequencies needed to obtain AUCs of 0.70–0.85 in addition to the 53 statistically significant genetic susceptibility variants (AUC = 0.69).

Risk allele Frequency	Number of extra genetic variants	AUC 0.70	AUC 0.75	AUC 0.80	AUC 0.85
0.05	1	1.2	2.3	5.1	9.0
	5	1.2	1.7	2.6	3.6
	20	1.1	1.4	1.7	2.1
	50	1.1	1.2	1.4	1.6
	100	1.1	1.2	1.3	1.4
0.30	1	1.2	1.9	3.0	4.9
	5	1.2	1.4	1.8	2.2
	20	1.1	1.3	1.4	1.6
	50	1.1	1.2	1.3	1.4
	100	1.1	1.2	1.3	1.4
0.50	1	1.2	1.8	3.2	5.3
	5	1.2	1.4	1.8	2.1
	20	1.1	1.3	1.4	1.5
	50	1.1	1.2	1.3	1.4
	100	1.1	1.2	1.3	1.4

NOTE: Odds ratios are presented as mean of 20 simulations each.

AUC: area under the receiver operating characteristic curve.

## Discussion

This study investigated the extent of MS prediction by genetic risk models, using empirical and simulation data on the most updated genetic information for MS. First, we showed that the predictive performance of testing multiple genes can be enhanced by using a combination of individual MS risk alleles. As expected, *HLA-DR* influences the ability to predict MS considerably due to its high OR. However, even without *HLA-DR* there was an increased, but small, risk for developing MS in people with 8 to 10 risk alleles. This underlines the current insight that multiple genes exert a small effect on developing MS on top of the major influence of *HLA-DR* [21], [22].

Next, after validating the genetic risk models with simulated genotype and MS status in a population of 100,000 individuals, we estimated that the predictive value as reflected in AUCs would be 0.66 when all 24 well replicated GWA derived polymorphisms were considered. Moreover, we showed that including the 29 novel risk genes increased the AUC only slightly to 0.69, illustrating that even more than doubling the number of risk SNPs does not increase the AUC sufficiently to make it useful in clinical practice. The AUC of 0.69 is comparable to other risk prediction models in MS [23], [24], [25]. In 2009, De Jager and colleagues investigated the prediction of 16 MS susceptibility loci using weighted genetic risk scores in three cohorts [23]. They demonstrated a consistent discriminatory ability in three independent samples (AUC varying 0.64–0.70). Gourraud and colleagues also investigated the aggregation of genetic MS risk markers in individuals by comparing multiple and single case families [24]. They showed that a greater genetic burden in siblings of MS patients was associated with an increased MS risk (OR 2.1, p = 0.001). However, the AUC for genetic burden differences between probands and siblings was only 0.57, indicating that the available genetic data is not sufficient to achieve case-control prediction of MS. They also used 16 MS susceptibility loci, partly matching with those of De Jager et al.

Before interpreting the clinical relevance of our findings, a methodological issue needs to be disclosed. We assumed that genetic variants inherited independently and that the combined effect of the genetic variants on disease risk followed a multiplicative risk model of independent effects (i.e., no statistical interaction terms were included in the model). Although so far no studies have demonstrated gene-gene interactions with MS risk, it is still possible that these will be discovered in future studies in larger populations. However, gene-gene interactions only improve the MS risk predictions if their effect sizes are substantially high (e.g., OR>5). When interaction effects are smaller, their effects on the predictive accuracy will be comparable with that of single gene effects, because by definition their frequencies are lower.

With the current model including 53 variants, we are still not able to differentiate with reasonable accuracy between individuals who will develop MS and those who will not (AUC 0.69). This makes our model not clinically useful. So the question is raised how to improve MS prediction.

We demonstrated in the simulation study that in order to obtain higher AUCs, a considerable number of additional common genetic variants or stronger associated variants with high ORs (table 4) need to be identified. The per-allele OR of the polymorphisms identified in GWA studies ranges from 1.08 to 2.1. When future GWA studies will identify polymorphisms with per-allele ORs around 1.1, the predictive ability of the genetic risk model can theoretically be improved beyond that of the existing models. Yet, even small improvements to 0.70 still require the discovery of 20 new statistically significant variants. Despite the increase it is still not clinically applicable. Because even in a disease that is readily treatable and even preventable like coronary heart disease (as presented in the Framingham Risk score) an AUC of about 0.80 is used [26]. For MS there is still no cure or preventive treatment available, and so a higher predictive accuracy is desirable to prevent false positives. We have shown that to pursue an AUC of 0.85, we have to include 50 new variants with ORs of 1.4 or a few common variants (minor allele frequency >30%) with high ORs (table 4). This may prove to be difficult, because the common genetic variants with high ORs may already have been identified, which would imply that even higher numbers of common genetic variants with relatively smaller ORs or many exceedingly rare variants (minor allele frequency <1%) with high ORs, will be needed. This seems not feasible. To note, unlike *HLA-DR* most of the genetic risk factors identified so far have only a slight effect on susceptibility to MS (with ORs that range from 1.1 to 1.2) [23]. However, more high risk genetic MS risk variants can be expected in near future [27]. With novel techniques such as next generation sequencing we can expect new rare variants with high ORs to be discovered [28]. This approach has already been proven successful in rare Mendelian disorders and can potentially also identify rare variants explaining the high recurrence rate of MS within families [29]. Also, this technique potentially allows us to find the causal variants for MS which will most likely have higher ORs than those found in GWA studies.

Another approach to improve MS prediction could be combining genetic with nongenetic risk factors such as infection with Epstein-Barr virus (EBV), smoking, and serum vitamin D concentrations [30]. It is likely that risk prediction models combined with nongenetic factors will perform better as ORs for SNPs tend to be smaller than ORs based on nongenetic factors (e.g. infectious mononucleosis [31]). De Jager and colleagues showed an enhanced discriminatory ability of 16 susceptibility genes by the inclusion of sex (AUC increasing from 0.70 to 0.74) and smoking and immune response to EBV (AUC increasing from 0.64 to 0.68). Others have performed studies combining the effects of *HLA-DR* and non-genetic factors like smoking and anti EBV serum levels [32], [33]. Also, integration of transcriptional, proteomics, and clinical factors will probably improve the prediction model and with that our understanding of MS genetics [34]. However, the added value of the SNPs might then be questioned. For other diseases it has been shown that the AUC does not improve a lot when adding SNPs to clinical risk factors. It should be noted though, that in these studies only small numbers of SNPs were added to the clinical risk factors.

Even if we can improve the prediction of MS in the future the question remains what the clinical implications of such predictive risk models would be. The discriminative accuracy that is required in preventive or clinical care depends on the goal of testing, the availability of (preventive) treatment, and the adverse effects of false-positive and false-negative test results.

Although the early results from GWA studies have not yet been used clinically, at least a partial goal of understanding the genetic basis of MS is to investigate the use of these variants to predict disease risk, so that environmental changes or therapeutic interventions can be initiated before the inflammatory demyelinating process progresses or even starts. Also, by better mapping the genetic of MS, we hope to improve our understanding of the pathofysiology of MS. This could help us finding better and new therapeutic drugs. By combining family history with a quantitative measure of genetic risk, a screening method might eventually be implemented that could identify clinically silent evidence of disease among first-degree relatives of MS patients, who have 20–50 times higher risk of developing MS [35]. However, the absolute risk is only 2–5% and therefore the models could be more useful in high risk populations with individuals who have had clinically isolated syndrome suggesting MS. These patients present with a neurological disability during their productive years of life and face the possibility of a chronic disease. Thus, they yearn for more clarity about their future. But also improving the risk prediction would enable us to distinguish individuals at risk to start early treatment for reducing the accumulation of neurological disability [36].

Given the possible clinical consequences of false-positivity within these patients, the required prediction AUCs for the pre-symptomatic diagnosis is considerably higher than an AUC intended for clinically isolated syndrome. It has been suggested that identified genetic variants have stronger effects in multiplex families [37]. It is of note that the ORs assessed up to now in GWA studies and validation studies are generally derived from datasets on sporadic cases. In a multiplex family setting, with potential stronger effects for individual risk variants, our estimates may prove to be conservative.

In conclusion, our analyses show that prediction of MS risk based on low susceptibility variants theoretically can improve prediction of disease when more variants are being discovered. However, the discriminatory ability in a clinical setting will be limited.

### Ethics approval

The empirical study was conducted with the approval of the institutional medical ethics committee of Erasmus MC, Rotterdam, The Netherlands.

## References

[1] Sadovnick AD, Baird PA, Ward RH (1988). Multiple sclerosis: updated risks for relatives.. Am J Med Genet.

[2] Ebers GC (2008). Environmental factors and multiple sclerosis.. Lancet Neurol.

[3] Hafler DA, Compston A, Sawcer S, Lander ES, Daly MJ (2007). Risk alleles for multiple sclerosis identified by a genomewide study.. N Engl J Med.

[4] Hoppenbrouwers IA, Aulchenko YS, Janssens AC, Ramagopalan SV, Broer L (2009). Replication of CD58 and CLEC16A as genome-wide significant risk genes for multiple sclerosis.. J Hum Genet.

[5] Hoppenbrouwers IA, Aulchenko YS, Ebers GC, Ramagopalan SV, Oostra BA (2008). EVI5 is a risk gene for multiple sclerosis.. Genes Immun.

[6] De Jager PL, Jia X, Wang J, de Bakker PI, Ottoboni L (2009). Meta-analysis of genome scans and replication identify CD6, IRF8 and TNFRSF1A as new multiple sclerosis susceptibility loci.. Nat Genet.

[7] Australia and New Zealand Multiple Sclerosis Genetics Consortium (2009). Genome-wide association study identifies new multiple sclerosis susceptibility loci on chromosomes 12 and 20.. Nat Genet.

[8] Hafler JP, Maier LM, Cooper JD, Plagnol V, Hinks A (2009). CD226 Gly307Ser association with multiple autoimmune diseases.. Genes Immun.

[9] The International Multiple Sclerosis Genetics Consortium and The Wellcome Trust Case Control Consortium (2011). Genetic risk and a primary role for cell-mediated immune mechanisms in multiple sclerosis.. Nature.

[10] Janssens AC, Moonesinghe R, Yang Q, Steyerberg EW, van Duijn CM (2007). The impact of genotype frequencies on the clinical validity of genomic profiling for predicting common chronic diseases.. Genet Med.

[11] Van Zitteren M, van der Net JB, Kundu S, Freedman AN, van Duijn CM (2011). Genome-based prediction of breast cancer risk in the general population: a modeling study based on meta-analyses of genetic associations.. Cancer Epidemiol Biomarkers Prev.

[12] Polman CH, Reingold SC, Edan G, Filippi M, Hartung HP (2005). Diagnostic criteria for multiple sclerosis: 2005 revisions to the “McDonald Criteria”.. Ann Neurol.

[13] Aulchenko YS, Hoppenbrouwers IA, Ramagopalan SV, Broer L, Jafari N (2008). Genetic variation in the KIF1B locus influences susceptibility to multiple sclerosis.. Nat Genet.

[14] Janssens AC, Aulchenko YS, Elefante S, Borsboom GJ, Steyerberg EW (2006). Predictive testing for complex diseases using multiple genes: fact or fiction?. Genet Med.

[15] Pepe MS, Gu JW, Morris DE (2010). The potential of genes and other markers to inform about risk.. Cancer Epidemiol Biomarkers Prev.

[16] Sackett DL, Haynes RB, Tugwell P (1985). Clinical epidemiology: a basic science for clinical medicine.

[17] Hanley JA, McNeil BJ (1982). The meaning and use of the area under a receiver operating characteristic (ROC) curve.. Radiology.

[18] Ihaka R GR (1996). R: A Language for Data Analysis and Graphics.. J Comput Graph Stat.

[19] Maller J, George S, Purcell S, Fagerness J, Altshuler D (2006). Common variation in three genes, including a noncoding variant in CFH, strongly influences risk of age-related macular degeneration.. Nat Genet.

[20] Van Hoek M, Dehghan A, Witteman JC, van Duijn CM, Uitterlinden AG (2008). Predicting type 2 diabetes based on polymorphisms from genome-wide association studies: a population-based study.. Diabetes.

[21] Oksenberg JR, Barcellos LF, Cree BA, Baranzini SE, Bugawan TL (2004). Mapping multiple sclerosis susceptibility to the HLA-DR locus in African Americans.. Am J Hum Genet.

[22] Lincoln MR, Montpetit A, Cader MZ, Saarela J, Dyment DA (2005). A predominant role for the HLA class II region in the association of the MHC region with multiple sclerosis.. Nat Genet.

[23] De Jager PL, Chibnik LB, Cui J, Reischl J, Lehr S (2009). Integration of genetic risk factors into a clinical algorithm for multiple sclerosis susceptibility: a weighted genetic risk score.. Lancet Neurol.

[24] Gourraud PA, McElroy JP, Caillier SJ, Johnson BA, Santaniello A (2011). Aggregation of multiple sclerosis genetic risk variants in multiple and single case families.. Ann Neurol.

[25] Sawcer S, Ban M, Wason J, Dudbridge F (2010). What role for genetics in the prediction of multiple sclerosis?. Ann Neurol.

[26] Wilson PW, D'Agostino RB, Levy D, Belanger AM, Silbershatz H (1998). Prediction of coronary heart disease using risk factor categories.. Circulation.

[27] Holm H, Gudbjartsson DF, Sulem P, Masson G, Helgadottir HT (2011). A rare variant in MYH6 is associated with high risk of sick sinus syndrome.. Nat Genet.

[28] Zeggini E (2011). Next-generation association studies for complex traits.. Nat Genet.

[29] Ng SB, Buckingham KJ, Lee C, Bigham AW, Tabor HK (2010). Exome sequencing identifies the cause of a mendelian disorder.. Nat Genet.

[30] Ascherio A, Munger K (2008). Epidemiology of multiple sclerosis: from risk factors to prevention.. Semin Neurol.

[31] Ramagopalan SV, Valdar W, Dyment DA, DeLuca GC, Yee IM (2009). Association of infectious mononucleosis with multiple sclerosis. A population-based study.. Neuroepidemiology.

[32] Simon KC, van der Mei IA, Munger KL, Ponsonby A, Dickinson J (2010). Combined effects of smoking, anti-EBNA antibodies, and HLA-DRB1*1501 on multiple sclerosis risk.. Neurology.

[33] Sundstrom P, Nystrom L, Jidell E, Hallmans G (2008). EBNA-1 reactivity and HLA DRB1*1501 as statistically independent risk factors for multiple sclerosis: a case-control study.. Mult Scler.

[34] Oksenberg JR, Baranzini SE, Sawcer S, Hauser SL (2008). The genetics of multiple sclerosis: SNPs to pathways to pathogenesis.. Nat Rev Genet.

[35] Compston A, Coles A (2008). Multiple sclerosis.. Lancet.

[36] Kieseier BC, Wiendl H, Leussink VI, Stuve O (2008). Immunomodulatory treatment strategies in multiple sclerosis.. J Neurol.

[37] D'Netto MJ, Ward H, Morrison KM, Ramagopalan SV, Dyment DA (2009). Risk alleles for multiple sclerosis in multiplex families.. Neurology.

